# Towards sustainable aquafeeds: Evaluating substitution of fishmeal with lipid-extracted microalgal co-product (*Nannochloropsis oculata*) in diets of juvenile Nile tilapia (*Oreochromis niloticus*)

**DOI:** 10.1371/journal.pone.0201315

**Published:** 2018-07-31

**Authors:** Pallab K. Sarker, Anne R. Kapuscinski, Ashley Y. Bae, Emily Donaldson, Alexander J. Sitek, Devin S. Fitzgerald, Oliver F. Edelson

**Affiliations:** Environmental Studies Program, Dartmouth College, Hanover, New Hampshire, United States of America; University of Illinois, UNITED STATES

## Abstract

Microalgae companies increasingly seek markets for defatted biomass that is left over after extracting omega-3 rich oil for human nutraceuticals and crude oil for fuels. Such a protein-rich co-product is a promising alternative to unsustainably sourced fishmeal in aquaculture diets. We report the first evaluation of co-product of the marine microalga *Nannochloropsis oculata* (*N*. *oculata* co-product) for replacing fishmeal in diets of Nile tilapia, a globally important aquaculture species. We conducted a nutrient digestibility experiment with *N*. *oculata* dried whole cells and *N*. *oculata* co-product, followed by an 84-day nutritional feeding experiment with *N*. *oculata* co-product. *N*. *oculata* co-product, more nutrient-dense than whole cells, had the highest digestibility for lysine, an essential amino acid that is often deficient in terrestrial crop meals; and for 20:5 n-3 EPA, making it a good option for EPA supplementation in tilapia feed. *N*. *oculata* co-product, despite containing higher amounts of protein than whole cells, had significantly lower digestibility for crude protein than whole cells. Apparent digestibility coefficients (ADC) of methionine were significantly lower in *N*. *oculata* co-product than in whole cells. The nutritional feeding experiment compared diets with *N*. *oculata* co-product that replaced fishmeal as follows: 0% replacement in reference diet (fishmeal as 7% of total diet) and test diets with 33%, 66% and 100% replacement of fishmeal (3%, 5.5%, and 8% of total diet, respectively). Results showed the 33% replacement diet yielded fish growth, feed conversion, and survival similar to the reference diet. Reduced digestibility and growth at greater *N*. *oculata* co-product inclusion levels may have been due to higher levels of anti-nutrients in co-product than whole cells. All diets yielded a n3:n6 ratio of tilapia fillet that is favorable for human consumption. Depositions of macro minerals and several trace elements in the fillet were not significantly different across diets. Thus, *N*. *oculata* co-product, when replacing 33% of fishmeal in tilapia feed, led to fish performance and flesh composition comparable to that of fish fed the reference diet, but its nutrient digestibility needs to be improved to achieve higher replacement levels.

## Introduction

Aquaculture, the world’s fastest growing food sector, made history in 2014 when the share of aquaculture production (c.74 million Mt) in the total food supply overtook global capture fisheries production (c.70 million Mt) for the first time [1]. Analysts predict that aquaculture production will account for two-thirds of global fish consumption by 2030 [2]). Nile tilapia (*Oreochromis niloticus*), a major aquaculture species and the focus of our current research, is predicted to be one of the two fastest growing aquaculture products in the next decade [3] and a key driver of US and global consumer demand for farmed fish [1]. As aquaculture expands, the proportion of farmed species fed commercial feeds is also increasing, representing approximately 70% of global aquaculture production in 2012 [4]. Developing sustainable feed ingredients in ways that add resilience to the global food system, therefore, is a major challenge for aquaculture [5].

Sustainable expansion of aquafeeds, among other things, necessitates finding alternatives to fishmeal and fish oil because of environmental, food security, and financial drawbacks of these ingredients. Aquaculture uses 70% of global supplies of fishmeal and fish oil, making it the largest consumer of these commodities [1,6]. Current levels of exploitation, and diversion of forage fisheries to produce fishmeal and fish oil undermine both marine biodiversity and human food security [7,8,9]. Diversion of small ocean fish to fishmeal and fish oil production erodes human food security because currently a quarter of the world’s commercially caught fish, 20 million tons of ocean fish, is directed away from human consumption every year, and instead, is used for fishmeal and fish oil production for other animal feeds [9]. More than 90 percent of these small, wild marine fish are considered food grade and could be directly consumed by humans, potentially creating an important source of nutrition for impoverished, food insecure people in developing countries [8,9]. Morever, aquafeed manufactures now face steep rises in fishmeal and fish oil prices, due to increased competition for these commodities from producers of human food supplements, pharmaceuticals and feeds for other animals [10].

These drawbacks of fishmeal and fish oil have motivated aquafeed producers to reduce their use of forage fish via partial substitution of fishmeal and fish oil with terrestrial plant ingredients [11,12]. This strategy is useful but insufficient to achieve nutritionally complete diet formulations. Methionine is the major limiting amino acid in soybean meal and lysine is the major limiting amino acid in cornmeal when these cropmeals are included in aquafeeds [13, 14]. Tilapia fed diets with soybean meal or cornmeal often show reduced growth due to these amino acid deficiencies [14, 15, 16]. Although amino acid supplementation of diets has increased performance of Nile tilapia [17], other investigators found that fish utilize synthetic amino acids less efficiently, and excrete more nitrogenous waste, than intact protein (protein-bound) [18, 19].

Overreliance on terrestrial crops also embroils aquaculture in concerns about massive diversion of crops from human consumption to animal feeds [5]. Tying aquaculture’s rapid growth to terrestrial staple crop feed inputs exacerbate existing environmental problems; i.e. the expansion of industrial terrestrial-crop (soy, corn) agriculture sectors which have had sizable contributions to both biodiversity loss and climate change [17,20,21]. Thus, unbalanced essential amino acids, low levels of long-chain polyunsaturated fatty acids, and high levels of anti-nutritional factors limit the inclusion rates of terrestrial plant ingredients, even in diets for omnivorous species like tilapia [17,22].

Marine microalgae show promise as alternative aquafeed ingredients that can improve environmental sustainability and human health benefits. Marine microalgae production offers several advantages over terrestrial crops: improved land and water-use efficiency due to higher yields per unit input, no need for freshwater and arable land, and lower greenhouse emissions [23,24,25]. Many marine microalgae species contain large amounts of DHA or EPA, omega-3 fatty acids that help prevent numerous human diseases [26]. Analysts predict that microalgae whole cells will quickly become a cost competitive substitute for fishmeal due to ongoing technological improvements that lower production costs for microalgae [27] although whole cells (US$1.65 per kg protein) currently cost more than fishmeal (US$1.30 per kg protein). Moreover, microalgal co-product, the protein-rich biomass left over after extracting oil for biofuel and nutraceutical products, is increasingly available and shows promise as a lower-cost replacement for conventional protein ingredients in tilapia feeds [28,29,30]. For the research presented in this paper, Qualitas Health, Inc. gave us a microalgae co-product, leftover from the company’s large-scale production of nutraceuticals. Integrating such industrial co-products into aquafeed could lower feed prices and provide an additional revenue stream from algae biomass and biofuel/ nutraceutical production. Several investigators showed the possibility of commercializing co-products as promising cost competitive protein-rich ingredients for animal feed [30,31,32][33].

In a previous study we successfully replaced all fish oil, in tilapia diets containing fishmeal, with a highly digestible, DHA-rich marine microalga, *Schizochytrium sp*. [33,34]. Now, this paper presents research studying the replacement of fishmeal with a protein-rich and EPA-rich co-product (lipid-extracted) of another marine microalga, *Nannochloropsis oculata* (*N*. *oculata*). Information about levels of nutrients and anti-nutrients and estimation of nutrient digestibility are the key steps to evaluate a new ingredient for growth of fish [35,36]. In the present study, therefore, we determined levels of nutrients and anti-nutrients of dried whole cells of *N*. *oculata* and *N*. *oculata* co-product; digestibility of their nutrients for tilapia; and effects of different proportions of fishmeal replacement with *N*. *oculata* co-product on fish growth and flesh n3:n6 ratios that are beneficial for human health.

## Materials and methods

The experimental design and fish use protocol were approved by the Institutional Animal Care and Use Committee (IACUC) of Dartmouth College. We euthanized the fish by single cranial pithing in the nutritional feeding experiment.

### Digestibility experiment

#### Feed formulation and preparation

We prepared a reference diet representing high quality tilapia feeds (Table 1) [33] and combined it with lyophilized *N*. *oculata* whole cells and co-product at a 7:3 ratio to produce two test diets following the apparent digestibility protocol of Cho et al. (1982)[37]. Qualitas Health Inc., which markets EPA-rich oil extracted from *N*. *oculata* as a human supplement [38] and seeks uses for tons of co-product from its large-scale *N*. *oculata* production, provided the *N*. *oculata* whole cells and co-product. In each diet we included an indigestible marker, also known as an insoluble ash marker, sipernat 50TM sourced from Evonik Degussa Corporation, Parsippany, NJ, USA [33]. We produced the diets by weighing and mixing oil and dry ingredients in a food mixer (Hobart Corporation, Tory, OH, USA) until full blended, then introduced water (330 ml kg^-1^ diet) into the mixture to attain a consistency appropriate for pelleting, and ran each diet through a meat grinder (Panasonic, MK-G20NR) to create 4 mm-diameter pellets. After pelleting, we dried the diets to a moisture content of 80–100 g kg^-1^ under a hood at room temperature for 12 h and then stored the finished feed in plastic containers at -20°C.

**Table 1:** Ingredient composition of the reference diet (g kg^-1^).

Ingredient (g/kg)	Diet		
	Reference	Test diet 1 (*Nanno*. whole cells)^3^	Test diet 2 (*Nanno*. co-product)
Fish meal	300	210	210
Soybean meal	170	119	119
Corn gluten meal	130	91	91
Fish oil	100	70	70
Wheat flour	280	196	196
Vitamin/mineral^1^	10	7	7
Sipernat 50^™^ (silicon dioxide marker)^2^	10	7	7
**Test ingredient**	**0**	**300**	**300**
Total	1000	1000	1000

^1^Vitamin/mineral premix (mg kg^-^1 dry diet unless otherwise stated):vitamin A (as acetate), 7500 IU kg^-^1 dry diet; vitamin D3 (as cholecalcipherol), 6000 IU kg^-^1 dry diet; vitamin E (as DL-a-tocopherylacetate), 150 IU kg^-^1 dry diet; vitamin K (as menadione Na-bisulphate), 3; vitamin B12 (as cyanocobalamin), 0.06; ascorbic acid (as ascorbyl polyphosphate), 150; D-biotin, 42; choline (as chloride), 3000; folic acid, 3; niacin (as nicotinic acid), 30; pantothenic acid, 60; pyridoxine, 15; riboflavin, 18; thiamin, 3; NaCl, 6.15; ferrous sulphate, 0.13; copper sulphate, 0.06; manganese sulphate, 0.18; potassium iodide, 0.02; zinc sulphate, 0.3; carrier (wheat middling or starch).

^2^Sipernat 50^™^: Source of acid insoluble ash comprised of 98.50% SiO_2_ with an average particle size of 50 μm.

^*3*^*Nanno* refers to *Nannochloropsis oculata*.

#### Experimental design and methods for digestibility study with N. oculata whole cells and co-product

The experiment employed a completely randomized design of three diets x four replicates (tanks). We used twelve static-water 114-L cylindro-conical tanks fitted with feces settling columns [33]. Juvenile Nile tilapia (*O*. *niloticus*) from Americulture Inc. (Animas, New Mexico, 88020, USA) were randomly assigned to tanks at a rate of 10 tilapia/tank, with a mean weight of 30 g/fish. We maintained a photoperiod cycle at 10 h light and 14 h dark and fed them the reference diet during a seven-day acclimation period. After randomly assigning the three diets to twelve tanks, we acclimated fish to experimental diets for seven days before initiation of feces collection. We hand-fed fish two times daily between 0930 and 1700h using restricted pair feeding to supply the same quantity of dietary nutrients to the groups [33,35]. We collected uneaten pellets directly after each feeding to minimize potential artifacts in the fecal samples, and we monitored water temperature, pH, and dissolved oxygen, ammonia and nitrite levels daily to maintain favorable conditions for tilapia. We replaced 50% of tank water, if total nitrogen or nitrite reached above 3.0 mg L -1 or 0.025 mg L -1 respectively, or twice per week. Water temperatures were kept within 27.0–28.0°C using a Finnex (HMA-100) 100watt immersion heater.

#### Fecal collection

We collected whole fish fecal strands twice daily, before the morning feeding and before the afternoon feeding, for 30 consecutive days, from an undisturbed fecal collection column affixed to the bottom of each tank [33]. We rinsed uneaten feed residues out of the fecal collection column after each feeding. In order to collect feces, we sealed off the bottom of the tank from the collector via gate valve, gently removed the full column, and then extracted settled feces using electronic pipetting (Eppendorf Easypet^®^ Serological Pipette Dispenser) before placing samples in 50 ml Falcon tubes (BD Falcon^™^). We allowed approximately 10 minutes to settle the fecal samples to the bottom of each Falcon tube before discarding supernatant water with the pipette and then froze the tubes at -20 °C. We pooled fecal samples by tank for the duration of the experiment. At the end of the experiment, we lyophilized, finely ground, and stored samples at -20°C for proximate, amino acid and fatty acid analysis.

#### Chemical analysis and calculations

We sent the four types of samples (*N*. *oculata* whole cells, *N*. *oculata* co-product, diets and feces) to New Jersey Feed Laboratory, Inc. (Ewing, NJ, USA), for the following analyses: moisture (Association of Official Analytical Chemists, AOAC 1995, no 930.15), crude protein (AOAC 990.03), lipid (AOAC 920.39), ash (AOAC 942.05), crude fiber (AOAC 1978.10), energy (automated oxygen bomb calorimeter), amino acids (high-performance liquid chromatography, HPLC analysis, via AOAC methods 994.12, 985.28, 988.15 and 994.12), and fatty acids (fatty acids methyl esters, FAME analysis, via AOAC method 963.22.). In addition, we analyzed acid-insoluble ash (AIA) in feed and feces, according to the methods of Naumann & Bassler (1976) and Keulen & Young (1977) [39,40]. We calculated apparent digestibility coefficients (ADC) for macronutrients, amino acids, fatty acids and energy of the test and the reference diets using the standard method as described by Cho et al. (1982) [37]:

ADC=1-(F/DxDi/Fi)

where: D = % nutrient (or kJ g^-1^ gross energy) of diet; F = % nutrient (or kJ g^-1^ gross energy) of feces; D_i_ = % digestion indicator (acid insoluble ashes; AIA) of diet; F_i_ = % digestion indicator (AIA) of feces.

We calculated the apparent digestibility of the microalgae as test ingredients as described by NRC [41]:

ADCtestingredient=ADCtestdiet+((ADCtestdiet–ADCref.diet)x(0.7xDref/0.3xDingredient))

where: D_ref_ is the percentage of nutrient or kcal/g gross energy in the reference diet, and D_ingredient_ is the percentage of nutrient or kcal/g gross energy in the ingredient.

Table 2 reports the proximate composition, gross energy, amino acid and fatty acid profiles of dried *N*. *oculata* whole cells and co-product. Table 3 reports the proximate composition, gross energy, amino acid, and fatty acid profiles of the reference diet, *N*. *oculata* whole cell diet, and *N*. *oculata* co-product diet.

**Table 2:** Proximate chemical composition, gross energy, essential amino acid and fatty acid profiles of the *N*. *oculata* whole cells and *N*. *oculata* co-product as test ingredients.

	Ingredients	
	*Nanno*. Whole cells	*Nanno*. co-product
Proximate composition (g kg^-1^as is)		
Crude protein	377	497
Lipid	66	48
Ash	267	86
Energy, kj g^-1^	1.9	1.3
Essential amino acids (g kg^-1^ in the weight of ingredient as is)		
Arginine	18.0	26.0
Lysine	26.0	27.0
Isoleucine	16.0	21.0
Leucine	32.0	42.0
Histidine	9.0	9.0
Methionine	7.0	10.0
Phenylalanine	17.2	24.3
Threonine	15.7	23.6
Tryptophan	4.6	4.5
Valine	22.6	29.0
Fatty acids fractions (g kg^-1^ of total fatty acids)		
Total SFA	187	287
Total MUFA	264	343
Total PUFA	549	371
20:5n-3 EPA	461	280
22:6n-3 DHA	ND	ND
Total n-3 PUFA	463	280
Total n-6 PUFA	79	87

SFA, saturated fatty acids; MUFA, monounsaturated fatty acids; PUFA, polyunsaturated fatty acids; EPA, eicosapentaenoic acid; DHA, docosahexaenoic acid.

ND, not detectable (<10 g kg^-1^ of total fatty acids).

*Nanno* refers to *Nannochloropsis oculata*.

**Table 3:** Proximate analysis, and gross energy, essential amino acid and fatty acid profiles of the reference and test diets.

	Diet		
	Ref	70%-Ref + 30%-*Nanno* whole cells	70%-Ref + 30%-*Nanno* co-product
Proximate composition (g kg^-1^as is as is)			
Crude protein	366	376	428
Lipid	114	108	103
Ash	77	151	90
Energy, kj g^-1^	13.7	13.5	14.3
Essential amino acid (g kg^-1^, in the weight of diet as is)			
Arginine	19.0	19.2	23.3
Lysine	19.7	22.4	24.4
Isoleucine	13.0	14.5	17.4
Leucine	34.2	35.3	41.2
Histidine	8.7	8.0	9.9
Methionine	7.9	8.0	9.0
Phenylalanine	17.7	18.3	22.0
Threonine	12.5	14.1	17.3
Tryptophan	2.5	3.2	3.2
Valine	15.4	18.2	21.9
Fatty acid fractions (g kg^-1^ of total fatty acids)			
Total SFA	293	293	319
Total MUFA	236	239	253
Total PUFA	469	469	428
20:5n-3 EPA	137	213	148
22:6n-3 DHA	117	75	87
Total n-3 PUFA	337	347	304
Total n-6 PUFA	99	89	89

Ref-reference diet; *Nanno* whole cells-*Nannochloropsis oculata* -whole cells; *Nanno* co-product-*Nannochloropsis oculata* coproduct

SFA, saturated fatty acids; MUFA, monounsaturated fatty acids; PUFA, polyunsaturated fatty acids; EPA, eicosapentaenoic acid; DHA, docosahexaenoic acid.

*Nanno* refers to *Nannochloropsis oculata*

#### Minerals and anti-nutrients analysis in N. oculata whole cells and co-product

*N*. *oculata* whole cell and co-product samples were analyzed in the Department of Earth Science at Dartmouth College for minerals analysis. 100 mg of each sample was acid digested in 0.5 ml 9:1 HNO3/HCl in an open vessel digestion with heating at 105 °C for 1 hr. The samples were diluted to 10 ml in DI water prior to analysis. All measurements were recorded gravimetrically. Digested samples were run by ICP-MS analysis using an Agilent 7700x with collision (He) and reaction (H_2_) gases. The methodology and quality control followed EPA method 6020a.

*N*. *oculata* whole cells and *N*. *oculata* co-product samples were analyzed for pectin, cellulose, and hemicellulose at the Complex Carbohydrate Research Center (University of Georgia, Athens, GA, USA) by glycosyl composition [42] and glycosyl linkage [43] analysis using combined gas chromatography/mass spectrometry (GC/MS). Trypsin inhibitor was analyzed in the New Jersey Feed Laboratory, Inc. (Ewing, NJ, USA). Trypsin inhibitor activity was determined via AOCS method Ba 12–75 [44]. This method is a spectrophotometric determination after an incubation of the sample. A defatted gram sample was extracted with 50 ml 0.01N NaOH for 3 hrs. An aliquot of this suspension was taken and diluted so that it inhibits approximately 40–60% of the trypsin available during the analysis. 2 mls of this suspension was incubated at 37 deg C for ten minutes after 2 mls of a trypsin solution and 5 mls of a BAPA (benzoyl-DL-arginine-p-niroanilide.HCl) solution are added to it. The reaction was terminated with the addition of 1 ml 30% acetic acid at the end of the ten-minute period. Sample and reagent blanks are also prepared. The resulting sample solutions were centrifuged 5 minutes at 10K ppm and measured at 410 nm on a spectrophotometer. The reported result was expressed in TI units per gram. We detected lectin content at Environmental Measurements Lab, Dartmouth. Lectin content was analyzed using an erythrocyte agglutinating assay that measured hemagglutinating units per milligram dried *N*. *oculata* whole cells and co-product (HU/mg). Solid lyophilized *N*. *oculata* whole cells and co-product were mixed overnight in phosphate buffered saline at 4°C. Trypsin treated rabbit erythrocytes were introduced into serially diluted concentrations of *N*. *oculata* co-product and whole solution and incubated at room temperature for 1 hour [45,46].

Table 4 reports crude fiber, pectin, cellulose, hemicellulose, trypsin inhibitor, and lectin levels of dried *N*. *oculata* whole cells and co-product. Table 5 reports macro minerals and trace elements in the dried *N*. *oculata* whole cells and co-product.

**Table 4:** Crude fiber, pectin, cellulose, hemicellulose, and trypsin inhibitor levels in the *Nannochloropsis oculata* whole cells and co-product.

Composition (as is)	Ingredients^1^	
	*Nanno* whole cells	*Nanno* co-product
Crude fiber (%)	0.8	3.7
Pectin (ug/mg)	ND^2^	ND
Cellulose (ug/mg)	ND	ND
Hemicellulose (ug/mg)	28.8	43.3
Lectin (HU/mg)	19.0	240.0
Trypsin inhibitor (TIU/g)	1000	2145

^1^*Nanno* refers to *Nannochloropsis oculata*

^2^ND, not detectable (<0.1 ug/400 ug).

**Table 5:** Macro minerals and trace elements in the *Nannochloropsis oculata* (*Nanno*) whole cells and co-product.

	Ingredients	
	*Nanno* whole cells	*Nanno* co-product
Macro minerals (%)		
Phosphorus	1.1 ± 0.0	1.4 ±0.0
Calcium	6.0 ± 0.3	0.8 ± 0.0
Magnesium	0.6 ± 0.0	0.6 ± 0.0
Potassium	1.0 ± 0.0	1.0 ± 0.0
Sulfur	0.03 ± 0.0	0.5 ± 0.0
Trace elements (mg kg−1)		
Copper	321.7 ± 18.6	61.0 ± 3.0
Iron	6098.8 ± 413.6	659.8 ± 14.9
Manganese	159.1 ± 4.2	91.6 ± 2.2
Selenium	3.0 ± 0.0	1.0 ± 0.0
Zinc	57.8 ± 2.4	47.0 ± 1.8
Boron	9.9 ± 1.0	1.0 ± 0.0
Aluminum	8100 ± 774	468.5 ± 53.7
Molybdenum	0.3 ± 0.5	0.4 ± 0.1
Arsenic	5.9 ± 0.0	0.2 ± 0.0
Mercury	ND	ND
Lead	15.6 ± 2.4	1.1 ± 0.1

ND, not detectable (<0.000 ug/g).

*Nanno* refers to *Nannochloropsis oculata*

### Nutritional feeding experiment

#### Diet formulation

Based on the results of the digestibility experiment, we incorporated *N*. *oculata* co-product into tilapia experimental diets for a nutritional feeding trial. We formulated four iso-nitrogenous (37% crude protein) and iso-energetic (12 kj/g) experimental diets in which *N*. *oculata* co-product replaces 0% (Nanno0), 33% (Nanno33), 66% (Nanno66), and 100% (Nanno100)% of the fish meal; thus *N*. *oculata* comprised 0%, 3%, 5.% and 8.0% of the diet by weight, respectively (Table 6). We used CMC as filler and varying levels of CMC did not affect pellet quality of experimental diets. Also, we did not observe any difference in pellet quality or fish feeding behavior among these experimental diets. The reference diet (Nanno0) had inclusion rates of fishmeal (7%) and fish oil (3.2%) that represent current levels in some high quality commercial tilapia feeds [47]. Feed preparation and analysis were performed as described for the digestibility experiment. At each level of *N*. *oculata* co-product replacement, the growth, feed efficiency, and nutritional quality of fish flesh were compared to that of fish on the control diet. Table 7 reports the fatty acid profiles of experimental diets.

**Table 6:** Formulation (g/100g diet) and proximate composition (%) and essential amino acids (% in the weight of diet) of four experimental diets for juvenile tilapia.

Ingredient	Diet			
	Nanno0	Nanno33	Nanno66	Nanno100
Fish meal*	7.0	4.69	2.38	0.0
*N*. *oculata* co-product	0.0	3.0	5.5	8.0
Corn gluten meal	28.0	28.0	28.0	28.0
Soybean meal	30.0	30.0	30.0	30.0
Wheat flour	25.0	25.0	25.0	25.0
CaH_2_PO_4_	0.75	0.75	0.75	0.75
Vitamin mix^1^	1.0	1.0	1.0	1.0
Mineral mix^2^	1.0	1.0	1.0	1.0
Fish oil	3.2	3.2	3.2	3.2
L-lysine HCL	0.0	0.0	0.0	0.25
Carboxymethyl cellulose	2.1	1.4	1.2	0.8
Choline chloride	2.0	2.0	2.0	2.0
Proximate compositions (%)				
Crude protein	37.7	37.6	37.5	38.3
Lipid	4.4	4.2	4.1	4.5
Ash	4.2	4.1	3.8	3.9
Crude fiber	1.6	1.5	1.4	1.4
Gross energy (Kj g-1)	2945	2907	2924	3161
Amino acids (% in the weight of diet as is)				
Arginine	1.8	1.8	1.8	1.8
Lysine	1.7	1.7	1.6	1.9
Isoleucine	1.4	1.5	1.5	1.7
Leucine	4.2	4.2	4.3	4.5
Histidine	0.9	0.9	0.9	0.9
Methionine	0.7	0.7	0.7	0.7
Cystine	0.6	0.6	0.6	0.7
Phenyle alanine	2.1	2.1	2.2	2.3
Threonine	1.4	1.4	1.4	1.5
Tryptophan	0.2	0.1	0.2	0.2
Valine	1.6	1.6	1.7	1.9

^1^Vitamin premix (mg kg-1 dry diet unless otherwise stated):vitamin A (as acetate), 7500 IU kg-1 dry diet; vitamin D3 (as cholecalcipherol), 6000 IU kg-1 dry diet; vitamin E (as DL-a-tocopherylacetate), 150 IU kg-1 dry diet; vitamin K (as menadione Na-bisulphate), 3; vitamin B12 (as cyanocobalamin), 0.06; ascorbic acid (as ascorbyl polyphosphate), 150; D-biotin, 42; choline (as chloride), 3000; folic acid, 3; niacin (as nicotinic acid), 30; pantothenic acid, 60; pyridoxine, 15; riboflavin, 18; thiamin, 3.

^2^Mineral premix (mg kg-1 dry diet unless otherwise stated):ferrous sulphate, 0.13; NaCl, 6.15; copper sulphate, 0.06; manganese sulphate, 0.18; potassium iodide, 0.02; zinc sulphate, 0.3; carrier (wheat middling or starch).

*Omega Protein, Inc. Houston, Texas 77042, as manufacturer specification, the guaranteed gross composition analysis: crude protein, 60%; crude fat, 6%; fiber, 2%.

**Table 7:** Fatty acid content (% of total fatty acids) of experimental diets.

Fatty acids (% of TFA)	Diet			
	Control (Nanno0)	Nanno33	Nanno66	Nanno100
14:00	6.6	6.5	6.4	6.9
15:00	0.5	0.6	0.7	0.9
16:00	19.5	19.6	19.1	19.0
17:00	0.5	0.5	0.6	0.7
18:00	3.4	3.4	3.4	3.8
20:00	0.3	0.4	0.4	0.4
22:00	0.3	0.4	0.3	0.4
24:00	0.3	0.3	0.3	0.5
Total SFA	31.3	31.7	31.3	32.6
16:1n9	ND	0.2	0.4	0.5
16:1n7	8.0	8.2	8.5	8.9
18:1n9	11.6	11.8	11.6	11.3
18:1n7	2.5	2.3	2.3	2.3
20:1n9	0.7	0.8	0.8	0.6
20:1n7	0.2	0.1	0.3	0.0
22:1n11	0.2	0.2	ND	0.3
22:1n9	0.4	0.5	0.4	1.0
24:1n9	0.2	0.4	0.3	0.2
Total MUFA	24.0	24.8	25.1	25.3
18:2n6	18.9	19.0	18.9	17.4
18:3n6	0.3	0.2	0.2	0.2
20:2n6	0.1	0.2	0.1	ND
20:3n6	0.2	0.2	0.2	0.3
20:4n6 ARA	1.0	1.1	1.2	1.3
22:4n6	ND	ND	ND	ND
22:5n6	0.4	0.3	0.4	0.4
Total n6 PUFA	21.0	20.9	21.0	19.6
18:3n3 ALA	2.1	2.1	2.1	2.0
18:4n3	1.3	1.2	1.2	1.2
20:3n3	ND	ND	ND	ND
20:4n3	0.8	0.7	0.7	0.7
20:5n3 EPA	8.7	8.6	8.8	9.0
22:5n3 DPA	1.7	1.7	1.6	1.5
22:6n3 DHA	5.8	5.4	5.0	4.8
Total n3 PUFA	21.0	20.0	20.1	19.8
Total PUFA	44.7	43.5	43.6	42.1
Total n6 LCPUFA	1.8	1.8	1.9	2.0
Total n3 LCPUFA	17.6	16.8	16.7	16.7
n3:n6 PUFA ratio	1.0	1.0	1.0	1.0

SFA, saturated fatty acids (sum of all fatty acids without double bonds); MUFA, monounsaturated fatty acids (sum of all fatty acids with a single bond); PUFA, polyunsaturated fatty acids (sum of all fatty acids with ≥2 double bonds); n6 PUFA, omega 6 polyunsaturated fatty acids (18:2, 18:3, 20:2, 20:3, 20:4, 22:4, 22:5); n6 LCPUFA, omega 6 long chain polyunsaturated fatty acids (20:2, 20:3, 20:4, 22:4, 22:5), n3 PUFA, omega 3 polyunsaturated fatty acids (18:3, 18:4, 20:3, 20:4, 20:5, 22:5, 22:6); n3 LCPUFA, omega 3 long chain polyunsaturated fatty acids (20:3, 20:4, 20:5, 22:5, 22:6); EPA, eicosapentaenoic acid; DHA, docosahexaenoic acid; ND, not detectable (<1% of total fatty acids).

#### Experimental design and methods to evaluate tilapia growth on N. oculata co-product diets

We used a completely randomized design of four diets x three replicates (tanks). Six hundred juvenile tilapia (mean initial weight 5 g) were randomly assigned to groups of 50 fish per tank, bulk-weighed and placed into twelve, 100 gal fish tanks, with each fish tank belonging to a recirculating aquaculture system module (RAS module). Stocking density (<0.25lb/gal, 80 gal of rearing water/tank) and water quality parameters in the RAS modules were maintained to ensure maximum growth of tilapia. Each RAS removed suspended solids and maintained acceptable ammonia-nitrogen and nitrite levels via bacterial nitrification in a BioClarifier bubble bead filter. We monitored water quality daily and did maintain favorable conditions for tilapia across all RAS modules and kept water temperature within 27.7 ± 0.09°C [34]. The range of values for other variables were pH 7.8 ± 0.2, dissolved oxygen 7.48 ± 0.18 mg L- 1, nitrite 0.09 ± 0.06 mg L- 1, and total ammonia nitrogen 0.24 ± 0.06 mg L- 1. We performed backwashes on each RAS module weekly to keep nitrates within acceptable levels <150 mg L -1.

We maintained all fish with the control diet for one week to adapt them to feeding and handling practices. We administered feed at a rate of 10% of body weight per day for the first 3 weeks, 8% of body weight until the 6th week, 6% of b.w. until the 9th week, and 4% of body weight until the 12th week [34,37,48]. Fish were hand fed three times a day for 12 weeks, and care was taken to ensure that feed waste was minimized. We bulk-weighed and counted the fish in each tank at 3-week intervals and adjust the feeding rate accordingly. For 24 hours prior to the weighing procedure we withheld food to avoid an increase in ammonia excretion due to handling.

#### Biological sampling and tissue collection

We weighed and sampled fish at the beginning of the experiment. Prior to commencing the feeding trial, we euthanized 20 fish from the stock, ground these fish into a homogeneous slurry, freeze-dried, reground, and stored at -20°C for whole-body proximate and fatty acid composition analysis. At week 12 of the experiment (terminus), 10 fish were removed from each tank and were immediately euthanized by single cranial pithing [34], and 5 fish filleted according to a standardized dorso-anterior landmark, packaged in sterile polythene bags (Whirl-pak, Naso, Fort Atkinson, Wisconsin), and stored frozen (-20°C) until fatty acid analysis. We freeze-dried the remaining 5 fish from each tank, finely ground and stored the samples at -20°C until whole-body carcass analysis.

#### Analytical procedure and calculation

We determined effects of different *N*. *oculata* co-product replacement levels on growth and survival by quantifying final weight, weight gain, weight gain percentage, feed conversion ratio (FCR), specific growth rate (SGR), protein efficiency ratio (PER), and survival rate. Calculations for these indices are as follows (Sarker 2016b; Sarker et al. 2012): Weight gain = (final weight—initial weight/initial weight) x 100; FCR, feed conversion ratio = feed intake/weight gain; protein efficiency ratio; SGR (%/day) = 100 x (ln final wet weight (g)—ln initial wet weight (g))/Time (days), PER = weight gain (g)/protein fed (g); and Survival rate (%) = (final number of fish/initial number of fish) x 100. Proximate, amino acids, and fatty acids of the samples were analyzed as described above for the digestibility experiment.

Diets and fillet samples were analyzed in the Department of Earth Science at Dartmouth College for minerals analysis as described above in the mineral analysis. Table 8 reports the macro minerals and trace elements content in the experimental diets.

**Table 8:** Macro minerals and trace elements in the experimental diets.

	Diet			
	Nanno0	Nanno33	Nanno66	Nanno100
Macro minerals (%)				
Phosphorus	0.7	0.6	0.6	0.6
Calcium	0.6	0.6	0.4	0.4
Magnesium	0.1	0.1	0.1	0.1
Potassium	0.8	0.8	0.8	0.8
Sulfur	0.4	0.4	0.4	0.4
Trace elements (mg kg−1)				
Copper	32.7	33.4	34.9	38.6
Iron	133.6	114.9	110.5	129.6
Manganese	85.1	80.5	81.7	85.2
Selenium	0.6	0.5	0.4	0.4
Zinc	96.9	88.2	76.6	104
Boron	14.2	12.0	12.2	11.4
Aluminum	94.9	67.6	63.9	58.8
Molybdenum	1.8	2.0	1.9	2.1
Arsenic	0.0	0.0	0.0	0.0
Mercury	ND	ND	ND	ND
Lead	0.3	0.1	0.1	0.1

ND, not detectable (<0.000 ug/g).

*Nanno* refers to *Nannochloropsis oculata*

We sent the diets, whole bodies, and fillets to New Jersey Feed Laboratory, Inc. (Ewing, NJ, USA) for the following types of analysis: proximate compositions, energy, amino acids, and fatty acids as described above in the digestibly experiment.

#### Statistical analysis

We conducted one-way analysis of variance (ANOVA) of apparent digestibility coefficients for macronutrients, fatty acids and amino acids in the reference and test diets, as well as for test ingredients. We then conducted ANOVA of growth performance and feed utilization parameters, whole body proximate composition, and fillet fatty acids composition and, when significant differences were found, compared the treatment means using Tukey’s test of multiple comparisons, with a 95% confidence interval. We carried out all statistical analyses using the IBM Statistical Package for the Social Sciences (SPSS) program for Windows (v. 21.0, Armonk, NY, USA).

## Results

### Digestibility of macronutrients and energy in diets

Table 9 compares ADC of nutrients, gross energy, essential amino acids and fatty acids across diets. We did not find significant differences between diets for the ADC of Crude protein (ranges from 68% to 71%), lipid (ranges from), and energy (ranges from 78 to 79%). The ADC of ash was significantly higher in the *N*. *oculata* co-product diet (43.2%) than in the *N*. *oculata* whole cells diet (27%), but reference (37.2%) and *N*. *oculata* co-product diet did not show any differences (Table 9).

**Table 9:** Apparent digestibility coefficients (ADC)* of nutrients, gross energy, essential amino acids and fatty acids in the reference diet and two test diets for tilapia.

	Diet			ANOVA	
	Ref	70%-Ref + 30%-*Nanno* whole cells	70%-Ref + 30%-*Nanno* co-product	F-value	P-values
ADC (%)					
Crude protein	71.4 ± 1.4	68.1 ± 3.4	69.6 ± 2.0	0.43	0.66
Lipid	86.5 ± 1.4^a^	80.0 ± 2.3^b^	80.0 ± 1.6^b^	5.2	0.02
Ash	37.2 ± 1.1^a^	26.7 ± 3.0^b^	43.2 ± 1.6^a^	15.7	<0.01
Energy	78.9 ± 1.8	73.1 ± 2.9	73.8 ± 1.7	2.04	0.18
Essential amino acids					
Methionine	66.6 ± 2.2	66.8 ± 2.5	64.1 ± 2.3	0.65	0.54
Lysine	76.0 ± 1.2	72.9 ± 3.4	75.1 ± 1.8	0.30	0.74
Tryptophan	80.0 ± 1.9^a^	83.1 ± 6.0^a^	70.6 ± 0.9^b^	8.57	0.02
Isoleucine	64.6 ± 1.1	66.5 ± 3.0	65.5 ± 2.2	0.11	0.89
Leucine	70.4 ± 1.2	68.6 ± 3.8	70.5 ± 2.0	0.12	0.88
Histidine	71.5 ± 0.5	66.1 ± 2.1	71.8 ± 2.7	1.93	0.20
Arginine	72.4 ± 1.2	69.3 ± 3.8	72.8 ± 1.8	0.49	0.62
Phenylalanine	71.2 ± 0.9	68.2 ± 3.6	69.2 ± 1.9	0.23	0.79
Threonine	68.4 ± 0.9	68.3 ± 3.7	65.9 ± 2.4	0.24	0.79
Valine	65.2 ± 1.5	67.3 ± 3.8	66.4 ± 2.3	0.12	0.88
Fatty acid fractions					
Total SFA	56.9 ± 1.0^b^	44.9 ± 4.6^c^	64.5 ± 1.2^a^	12.16	<0.01
Total MUFA	70.0 ± 2.0^a^	58.5 ± 4.2^b^	64.1 ± 2.1^ab^	4.33	0.03
Total PUFA	82.7 ± 1.9^a^	75.2 ± 2.2^ab^	73.4 ± 2.1^b^	5.63	<0.01
20:5n-3 EPA	86.3 ± 1.9^a^	79.0 ± 1.6^ab^	70.5 ± 2.3^b^	15.03	<0.01
22:6n-3 DHA	87.7 ± 1.9^a^	78.5 ± 2.2^b^	81.3 ± 1.9^ab^	5.78	0.02
Total n-3 PUFA	86.2 ± 1.9^a^	77.7 ± 2.2^ab^	72.3 ± 2.1^b^	7.93	<0.01
Total n-6 PUFA	72.4 ± 1.8	65.2 ± 3.3	65.2 ± 2.1	2.67	0.12

SFA, saturated fatty acids; MUFA, monounsaturated fatty acids; PUFA, polyunsaturated fatty acids; EPA, eicosapentaenoic acid; DHA, docosahexaenoic acid.

* Means of ADC of nutrients in reference and test diets for tilapia. Mean values across the row not sharing a common superscript were significantly different as determined by Tukey’s HSD test, P<0.05.

*Nanno* refers to *Nannochloropsis oculata*

The ADCs of all essential amino acids, except for tryptophan, were not significantly different among the co-product, whole cells, and reference diet. We found significantly higher ADC of tryptophan in the *N*. *oculata* whole cell diet (83%) than *N*. *oculata* co-product diet (71%) but no significant difference between *N*. *oculata* whole cell diet and reference diet (80%).

The ADC of most of the major fatty acid fractions were significantly different among test diets (Table 9). We found significantly higher ADC of saturated fatty acids (SFA) in the *N*. *oculata* co-product diet (65%) than *N*. *oculata* whole cells (45%) and reference diet (57%); and lowest ADC value of total MUFA in the *N*. *oculata* whole cells diet compared to reference diet. We found significantly higher ADCs of total PUFA, n3 PUFA, and 20:5n3 EPA in reference diet than *N*. *oculata* co-product diet; and no difference between reference and *N*. *oculata* whole cells diet. We observed significantly higher ADC of 20:5n3 EPA in fish fed the reference diet (86%) than the *N*. *oculata* whole cells diet (79%); but no difference between reference and *N*. *oculata* co-product (71%). We did not detect significant differences between *N*. *oculata* co-product and reference diet for the ADC of 22:6n3 DHA; and detected the lowest value in *N*. *oculata* whole cells.

#### Digestibility of macronutrients and energy in microalgal ingredients

Table 10 presents calculated digestibility of nutrients, energy, essential amino acids and fatty acids in microalgal ingredients. We detected significantly higher ADC values for crude protein and energy in the *N*. *oculata* whole cells (81% and 80%) than the *N*. *oculata* co-product (74% and 73%). We did not detect significant differences between *N*. *oculata* whole cells and *N*. *oculata* co-product for the ADC of lipid. We detected the lowest ADC value of ash in the *N*. *oculata* whole cells (24%), compared with the *N*. *oculata* co-product (54%).

**Table 10:** Test ingredient apparent digestibility coefficients (ADC_test_ingredient)* of nutrients, gross energy, essential amino acids and fatty acids in *Nannochloropsis oculata*–*whole cells* (*Nanno* whole cells) and *Nannochloropsis oculata*–co-product (*Nanno* co-product) for tilapia.

	Ingredients		ANOVA	
	*Nanno* whole cells	*Nanno* co-product	F-value	P-values
ADC (%)				
Crude protein	81.1 ± 6.7^a^	73.5 ± 0.0^b^	49.07	0.02
Lipid	64.2 ± 8.3	60.6 ± 8.3	0.07	0.8
Ash	23.9 ± 4.2^b^	53.8 ± 4.3^a^	18.12	<0.01
Energy	80.0 ± 0.6^a^	72.8 ± 3.5^b^	11.84	0.02
Essential amino acids				
Methionine	88.1 ± 3.1^a^	64.1 ± 2.3^b^	35.7	<0.01
Lysine	75.8 ± 14.5	81.5 ± 2.4	0.75	0.45
Tryptophan	86.5 ± 0.6^a^	56.1 ± 3.3^b^	47.72	<0.01
Isoleucine	79.8 ± 15.1	73.6 ± 11.3	0.11	0.75
Leucine	78.3 ± 16.6	81.3 ± 11.4	0.01	0.9
Histidine	74.0 ± 0.1^b^	76.7 ± 0.4^a^	0.98	0.39
Arginine	71.4 ± 9.5	83.0 ± 10.8	0.57	0.5
Phenylalanine	72.5 ± 19.0	74.0 ± 9.7	0	0.94
Threonine	66.6 ± 13.6	60.5 ± 6.1	0.18	0.68
Valine	77.7 ± 12.3	73.4 ± 8.0	0.08	0.78
Fatty acid fractions				
Total SFA	39.6 ± 2.0^b^	82.2 ± 3.3^a^	9.68	0.03
Total MUFA	57.1 ± 2.3	54.8 ± 7.3	0.08	0.79
Total PUFA	74.1 ± 0.8^a^	58.1 ± 0.7^b^	185.97	<0.01
20:5n-3 EPA	94.0 ± 7.3	96.9 ± 4.3	0.08	0.78
22:6n-3 DHA	ND	ND		
Total n-3 PUFA	63.3 ± 7.0	57.6 ± 0.1	4.41	0.08
Total n-6 PUFA	70.9 ± 3.1^a^	55.3 ± 0.7^b^	23.12	0.04

SFA, saturated fatty acids; MUFA, monounsaturated fatty acids; PUFA, polyunsaturated fatty acids; EPA, eicosapentaenoic acid; DHA, docosahexaenoic acid.

* Means of ADC of nutrients in reference and test diets for tilapia. Mean values across the row not sharing a common superscript were significantly different as determined by Tukey’s HSD test, P<0.05.

ND, not detectable (<1% of total fatty acids).

*Nanno* refers to *Nannochloropsis oculata*

Regarding amino acids, ADCs of methionine and tryptophan were significantly lower in the *N*. *oculata* co-product (64% and 56%) than in the *N*. *oculata* whole cells (88% and 87%). We found the lowest ADC values of isoleucine, threonine, and valine in *N*. *oculata* co-product, compared with Na whole cells. However, we found higher ADC values of arginine, histidine, lysine, leucine, and phenylalanine in *N*. *oculata* co-product than in *N*. *oculata* whole cells (Table 10).

We detected significantly different ADCs of all fatty acid fractions among the microalgal ingredients were significantly different, except for total MUFA, 20:5n3 EPA, and total n-3 PUFA (Table 10). The ADCs of total PUFA and total n6-PUFA were significantly lower in *N*. *oculata* co-product (58% and 55%) than in *N*. *oculata* whole cells (74% and 71%). We observed the highest ADC value of total SFA in *N*. *oculata* co-product (82%) when compared with *N*. *oculata* whole cells (40%).

### Nutritional feeding experiment

Table 11 summarizes the results of the 84-day growth experiment we conducted to test effects of replacing different percentages of dietary fishmeal with *N*. *oculata* co-product. Results showed comparable final weight, weight gain, percent weight gain, specific growth rate (SGR), and protein efficiency ratio (PER) Nanno0 diet and Nanno33 diet; and lower performance at higher *N*. *oculata* co-product inclusion levels. We found no significant differences in feed conversion (FCR) and feed intake (FI) among the diets. Tilapia appeared healthy at the end of the experiment with survival rates ranging from 94 to 98% among all diet groups, with no significant difference in survival rates. Also, the *N*. *oculata* whole cell and *N*. *oculata* co-product diets showed high palatability to tilapia, who fed equally well as on *N*. *oculata*-based diets as the reference diet.

**Table 11:** Initial weight, final weight, weight gain, percentage weight gain, feed conversion ratio (FCR), specific growth rate (SGR), protein efficiency ratio (PER), feed intake, and survival rate of tilapia fed experimental diets.

	Diet^1^				ANOVA	
	Nanno0	Nanno33	Nanno66	Nanno100	*F* value	*P* value
Initial weight (g)	1.97 ± 0.1	1.98 ± 0.1	2.0 ± 0.1	2.0 ± 0.0	2.11	0.17
Final weight (g)	33.83 ± 2.38^a^	28.60 ± 0.9^a^	24.30 ± 1.34^b^	25.93 ± 1.49^b^	6.72	0.01
Weight gain (g)^2^	31.91 ± 2.37^a^	26.63 ± .90^a^	22.31 ± 1.34^b^	23.93 ± 1.48^b^	6.8	0.01
Weight gain (%)^3^	1616.6 ± 112.7^a^	1346.3 ± 47.3^a^	1117.6 ± 67.9^b^	1198.0 ± 71.9^b^	7.8	<0.01
FCR^4^	1.12 ± 0.08	1.26 ± 0.03	1.58 ± 0.17	1.55 ± 0.11	4.2	0.06
SGR^5^	3.38 ± .08^a^	3.18 ± 0.04^a^	2.97 ± 0.07^b^	3.05 ± 0.7^b^	8.0	<0.01
PER^6^	2.41 ± 0.18^a^	2.12 ± 0.5^a^	1.72 ± 0.17^b^	1.74 ± 0.12^b^	5.4	0.02
Feed intake (g/fish)	35.40 ± 0.7	33.54 ± 0.3	34.78 ± 1.7	36.88 ± 1.8	1.1	0.39
%Survival rate^7^	98.00 ± 4.16	94.67 ± 4.37	96.70 ± 0.67	96.0 ± 3.06	0.78	0.53

Values are means of ±SE of three replicate groups (n = 3).

^1^Mean values not sharing a superscript letter in the same row differ significantly (P < 0.05).

^2^Weight gain (g) = final wet weight–initial wet weight.

^3^Weight gain (%) = (final wet weight–initial wet weight/initial wet weight) x 100.

^4^FCR, feed conversion ratio = feed intake/ weight gain.

^5^Specific growth rate SGR (%/day) = (ln final wet weight (g)–ln initial wet weight (g)) / Time (days).

^6^PER, protein efficiency ratio = weight gain (g)/protein fed (g).

^7^Survival rate (%) = (Final number of fish / Initial number of fish) x 100.

*Nanno* refers to *Nannochloropsis oculata*

Whole body proximate composition of Nile tilapia fillets did not significantly differ among dietary treatments. This included moisture, crude protein, ash and total lipid. The crude protein contents ranged from 13% to 14% among the five dietary treatments. The lipid contents were 7% among the five dietary treatments.

Table 12 shows no significant differences in fillet macro mineral composition but some differences in fillet trace element composition among dietary treatments. With respect to trace elements, concentrations of iron in the fillet of tilapia fed the Nanno66 and Nanno100 had significantly higher levels compared to fish fed the Nanno0 and Nanno33 diets. The fillet zinc concentration of Nile tilapia fed Nanno100 had significantly higher levels compared to fish fed the other diets. Concentrations of manganese in the fillet were significantly lower in fish fed Nanno100 compared to Nanno0, Nanno33, and Nanno66 diets. Concentrations of Cu, S, and, Mo in whole body and fillet were not significantly different (*p*>0.01) among all the diets. Several trace elements, for example, B, Al, Hg, and, Pb in the fillet, were below instrument detection level among all treatments.

**Table 12:** Macro minerals and trace elements content (wet weight basis) of fillets from Nile tilapia after 84 days on the experimental diets.

	Diet				ANOVA	
	Nanno0	Nanno33	Nanno66	Nanno100	*F*	*P*
Macro minerals (%)						
Phosphorus	0.7 ± 0.0	0.7 ±0.0	0.7 ±0.0	0.7 ±0.0	1.63	0.21
Calcium	0.4 ± 0.3	0.2 ± 0.1	0.3 ±0.1	0.3 ±0.0	2.89	0.06
Magnesium	0.1 ± 0.0	0.1 ± 0.0	0.1 ±0.0	0.1 ±0.0	0.12	0.94
Potassium	1.1 ± 0.2	1.0 ± 0.3	1.1 ± 0.4	1.1 ±0.3	0.65	0.58
Sulfur	0.6 ± 0.0	0.6 ± 0.0	0.7 ±0.0	0.7 ±0.0	0.64	0.56
Trace elements (mg kg−1)						
Copper	1.7 ± 0.2	1.4 ± 0.1	1.7 ± 0.1	1.6 ± 0.0	2.84	0.06
Iron	13.5 ± 0.5^ab^	11.4 ± 0.4^b^	14.1 ± 0.8^a^	14.0 ± 0.8^a^	4.13	0.02
Manganese	1.3 ± 0.1^a^	1.1 ± 0.1^a^	1.2 ± 0.1^a^	0.8 ± 0.1^b^	5.24	<0.01
Selenium	0.6 ± 0.02	0.5 ± 0.0	0.6 ± 0.0	0.5 ± 0.0	2.2	0.11
Zinc	21.5 ± 1.3^b^	18.6 ± 0.8^b^	20.2 ± 0.9^b^	28.1 ± 1.6^a^	11.78	<0.01
Arsenic	0.0 ± 0.0	0.0 ± 0.0	0.0 ± 0.0	0.0 ± 0.0		
Boron	ND	ND	ND	ND		
Aluminum	ND	ND	ND	ND		
Mercury	ND	ND	ND	ND		
Lead	ND	ND	ND	ND		
Molybdenum	0.1 ± 0	0.1 ± 0.0	0.1 ± 0	0.1 ± 0.0	3.5	0.06

Mean values are the mean of 3 replicates (±SE) with each replicate involving pooled whole tissues of 5 fish. Values across the row not sharing a common superscript were significantly different as determined by Tukey’s HSD test, P<0.05. ND, not detectable (<0.000 ug/g).

*Nanno* refers to *Nannochloropsis oculata*

The fatty acid profile of the fillet tissue of tilapia fed the experimental diets reflected the dietary fatty acid content of the corresponding feed (Table 13). The concentrations of total n3 PUFA, n6 PUFA, 20:5n3 EPA, 22:6n3 DHA, total PUFA, and total n3 LC PUFA were not significantly different (*p*>0.01) in any of the diets. Similarly, the total saturated fatty acid (SFA), composition of SFA fractions, total MUFA, and most MUFA fractions did not differ across treatments. However, the concentrations of 16:1n7 were significantly affected by dietary treatments, as fish fed the Nanno100 diet displayed the highest concentrations, which was directly related to the 16:1n7 content in experimental diets (Table 7). Amounts of major n3 and n6 PUFA deposition in the fish fillet (mg/g fillet) were not significantly different among the four dietary treatments (Table 14).

**Table 13:** Fatty acid (% of total fatty acids) content of fillets from Nile tilapia after 84 days on the experimental diets; average ± SE for 3 replicates per diet (pooled whole tissues of 5 fish/replicate)§.

Final Fillet (%TFA) ± SE						
Fatty acids (% TFA)	Fillet				F value	P value
	Control	Nanno33	Nanno66	Nanno100		
14:00	4.9 ± 0.1	4.8 ± 0.1	4.7 ± 0.1	4.9 ± 0.1	1.14	0.38
15:00	0.4 ± 0.00	0.4 ± 0.0	0.4 ± 0.1	0.4 ± 0.00	0.81	0.52
16:00	24.7 ± 0.2	24.7 ± 0.1	24.8 ± 0.5	24.8 ± 0.0	0.08	0.96
17:00	0.4 ± 0.0	0.6 ± 0.2	0.3 ± 0.0	0.4 ± 0.00	0.68	0.58
18:00	5.7 ± 0.1	5.9 ± 0.1	5.5 ± 0.2	5.5 ± 0.1	2.3	0.14
20:00	0.3 ± 0.00	0.3 ± 0.0	0.3 ± 0.0	0.3 ± 0.0	0.19	0.89
22:00	0.1 ± 0.1	ND	ND	ND		
24:00:00	ND	ND	ND	ND		
Total SFA	36.6 ± 0.1	36.7 ± 0.3	36.3 ± 0.3	36.3 ± 0.2	1.5	0.26
16:1n9	0.9 ± 0.1	0.9 ± 0.1	0.9± 0.1	0.8 ± 0.0	0.93	0.46
16:1n7	7.0 ± 0.3^b^	7.1 ± 0.1^b^	7.5 ± 0.2^a^	7.8 ± 0.2^a^	4.16	0.04
18:1n9	23.1 ± 0.4	23.3 ± 0.1	23.4 ± 0.5	23.7 ± 0.2	0.56	0.65
18:1n7	4.1 ± 0.0	4.1 ± 0.1	4.1 ± 0.0	4.1 ± 0.0	1.01	0.43
20:1n9	1.5 ± 0.1	1.5 ± 0.0	1.3 ± 0.0	1.3 ± 0.0	3.85	0.06
20:1n7	0.1 ± 0.1	0.1 ± 0.0	0.1 ± 0.1	0.2 ± 0.1	0.06	0.97
22:1n9	ND	ND	ND	ND		
24:1n9	ND	ND	ND	ND		
Total MUFA	37.2 ± 0.5	37.5 ± 0.3	37.9 ± 0.6	38.2 ± 0.2	1.32	0.33
18:2n6	9.5 ± 0.2	9.3 ± 0.1	9.6 ± 0.4	9.7 ± 0.1	0.68	0.58
18:3n6	0.6 ± 0.00	0.7 ± 0.0	0.7± 0.1	0.6 ± 0.0	1.14	0.38
20:2n6	0.3 ± 0.0	0.4 ± 0.0	0.7 ± 0.0	0.3 ± 0.0	0.67	0.58
20:3n6	0.8 ± 0.0	0.8 ± 0.0	0.7± 0.0	0.7 ± 0.0	0.71	0.56
20:4n6 ARA	1.7 ± 0.1	1.6 ± 0.0	1.4 ± 0.1	1.4 ± 0.0	3.25	0.08
22:4n6	ND	ND	ND	ND		
22:5n6	0.7 ± 0.0	0.7 ± 0.0	0.6 ± 0.0	0.7 ± 0.0	1.23	0.35
Total n6 PUFA	13.6 ± 0.2	13.4 ± 0.1	13.5 ± 0.6	13.4 ± 0.0	0.07	0.97
18:3n3 ALA	0.8 ± 0.0	0.8 ± 0.0	0.8 ± 0.0	0.8 ± 0.0	1.01	0.43
18:4n3	0.4 ± 0.0	0.4 ± 0.0	0.4 ± 0.1	0.4 ± 0.0	0.18	0.90
20:3n3	ND	ND	ND	ND		
20:4n3	0.3 ± 0.0	0.3 ± 0.0	0.3 ± 0.0	0.3 ± 0.0	0.85	0.50
20:5n3 EPA	1.2 ± 0.0	1.2 ± 0.0	1.2 ± 0.0	1.2 ± 0.0	0.57	0.64
22:5n3	2.8 ± 0.1	2.7 ± 0.0	2.9 ± 0.1	2.9 ± 0.0	1.69	0.24
22:6n3 DHA	6.1 ± 0.3	5.8 ± 0.1	5.7 ± 0.2	5.4 ± 0.1	1.83	0.21
Total n3 PUFA	11.7 ± 0.2	11.4 ± 0.1	11.5 ± 0.2	11.2 ± 0.2	0.57	0.64
Total PUFA	26.3 ± 0.6	25.8 ± 0.2	26.1 ± 0.8	25.4 ± 0.2	0.52	0.67
Total n6 LCPUFA	3.5 ± 0.2	3.4 ± 0.1	3.1 ± 0.2	3.2 ± 0.0	2.83	0.10
Total n3 LCPUFA	10.5 ± 0.5	10.2 ± 0.2	10.3 ± 0.2	10.0 ± 0.2	0.61	0.62
n3/n6 PUFA ratio	0.9 ± 0.0	0.9 ± 0.0	0.9 ± 0.0	0.8 ± 0.0	0.17	0.91
n3/n6 LCPUFA ratio	3.0 ± 0.1	3.0 ± 0.1	3.3 ± 0.2	3.2 ± 0.0	2.22	0.16
20:5n3 EPA/20:4n6 ARA	1.4 ± 0.5	1.3 ± 0.1	1.2 ± 0.1	1.2 ± 0.0	1.46	0.29

SFA, saturated fatty acids (sum of all fatty acids without double bonds); MUFA, monounsaturated fatty acids (sum of all fatty acids with a single bond); PUFA, polyunsaturated fatty acids (sum of all fatty acids with ≥2 double bonds); n6 PUFA, omega 6 polyunsaturated fatty acids (18:2, 18:3, 20:2, 20:3, 20:4, 22:4, 22:5); n6 LCPUFA, omega 6 long chain polyunsaturated fatty acids (20:2, 20:3, 20:4, 22:4, 22:5), n3 PUFA, omega 3 polyunsaturated fatty acids (18:3, 18:4, 20:3, 20:4, 20:5, 22:5, 22:6); n3 LCPUFA, omega 3 long chain polyunsaturated fatty acids (20:3, 20:4, 20:5, 22:5, 22:6); EPA, eicosapentaenoic acid; DHA, docosahexaenoic acid, and n3:n6 ratio calculated for total n3 PUFA: total n6 PUFA. Mean values across the row not sharing a common superscript were significantly different as determined by Tukey’s HSD test, P<0.05. ND, not detectable (<0.1% of total fatty acids).

^§^ in many cases ± 0.0 (S.E) values are rounding error.

*Nanno* refers to *Nannochloropsis oculata*

**Table 14:** Amounts of lipid and major n3 and n6 PUFA in the fillet (wet weight basis) of Nile tilapia fed experimental diets for 84 days; average ± SE for 3 replicates per diet (pooled whole tissues of 5 fish/replicate).

Fillet PUFA (mg/g) ± SE						
Fatty acids (mg/g fillet)	Fillet				ANOVA	
	Control (Nanno0)	Nanno33	Nanno66	Nanno100	F value	P value
18:2n6 LA	2.7 ± 0.1	2.9 ± 0.0	3.4 ± 0.2	3.2 ± 0.2	1.82	0.22
20:4n6 ARA	0.5 ± 0.0	0.5 ± 0.0	0.5 ± 0.0	0.4 ± 0.0	0.61	0.62
18:3n3 ALA	0.2 ± 0.0	0.3 ± 0.0	0.3 ± 0.0	0.3 ± 0.0	1.4	0.31
20:5n3 EPA	0.3 ± 0.0	0.4 ± 0.0	0.4 ± 0.0	0.4 ± 0.0	0.99	0.44
22:6n3 DHA	1.7 ± 0.0	1.8 ± 0.0	2.0 ± 0.0	1.8 ± 0.0	1.38	0.31

LA, linoleic Acid; ARA, arachidonic acid; ALA, alpha linolenic acid; EPA, eicosapentaenoic acid; DHA, docosahexaenoic acid. Mean values across the row not sharing a common superscript were significantly different as determined by Tukey’s HSD test, P<0.05.

*Nanno* refers to *Nannochloropsis oculata*

## Discussion

Our digestibility study found that *N*. *oculata* co-product is a highly nutrient-dense feedstuff compared to *N*. *oculata* whole cell and offers the highest digestibility of lysine, an essential amino acid that is often deficient in terrestrial crop meals used in aquafeeds. We also detected the highest 20:5 n-3 EPA digestibility in co-product (97%), indicating that it would be a good candidate for EPA supplementation in tilapia diet formulation. Results show that, even though *N*. *oculata* co-product contains higher amounts of protein compared to *N*. *oculata* whole cells, co-product had significantly lower digestibility values for crude protein than whole cells. Results from our nutritional feeding experiment showed, compared to the control, good growth, feed conversion, and survival of tilapia fed a diet replacing 33% of fish meal with *N*. *oculata* co-product and decreased fish performance at higher inclusion levels. These findings indicate a need to improve nutrient digestibility in order to achieve a higher replacement level. This is the first report of effects of different inclusion levels of a micro-algal co-product in tilapia diets.

Our digestibility results show that *N*. *oculata* co-product contained higher amounts of protein compared to *N*. *oculata* whole cells (50% vs. 38%) but had significantly lower digestibility values for crude protein than the whole cells (74% vs. 81%). Our reported ADC of crude protein in the *N*. *oculata* whole cells ingredient is comparable to levels reported for microalgal whole cells in tilapia, including *Chlorella* sp. (80%) and *Schzochytrium* sp. (82%) [33], and *Anabaena* sp.[49]. The ADC of crude protein in the *N*. *oculata* whole cells and *N*. *oculata* co-product ingredients was lower than some other commonly used proteins sources, *Spirulina*, fishmeal, and soybean meal. And it was higher than the ADC of crude protein in some crops used as protein and carbohydrates sources, including corn, broken rice, sorghum, wheat middlings and rice bran, which have ranged from 57% (sorghum) to 73% (corn) in tilapia [33,50].

Our digestibility results are promising with respect to higher digestibility of lysine, which is one of the most deficient amino acids in terrestrial protein used for aquaculture feed. Algal biomass could be a desirable source of EAAs in aquaculture feeds, as well as other animal feeds [13, 14]. If algae biomass is produced and made available in larger quantities, it could become cost competitive or cheaper than crystalline amino acid supplementation, provided it shows adequate digestibility in the farmed animals. For Nile tilapia, we observed significantly lower ADCs of methionine in *N*. *oculata* co-product than in *N*. *oculata* whole cells (64.1% vs. 88.1%) but higher lysine digestibility in *N*. *oculata* co-product (81%) compared to *N*. *oculata* whole cells (76%). Future research is needed to identify why ADCs for both amino acids differed between whole cells and co-product. These *N*. *oculata* co-product values are similar to those reported in canola meal for hybrid tilapia, specifically ADC of 64% for methionine and 80% for lysine [51]; and somewhat lower than our previous reported values of ADCs >90.0% in *Spirulina* and *Schizochytrium spp*. for methionine and lysine in Nile tilapia [33].

Test ingredient digestibility results for fatty acids suggest that *N*. *oculata*. co-product would be a good candidate for EPA supplementation in tilapia diet formulation and energy provided by SFA. First, we detected high digestibility for 20:5 n-3 EPA for *N*. *oculata* co-product (97%) and whole cell (94%), comparable to our previously reported DHA and EPA digestibility (>94%) for *Schizochytrium* sp. [33]. Second, we found the highest total saturated fatty acid, SFA (82%) digestibility in the *N*. *oculata* co-product, which is higher than our findings for *Schizochytrium* (52%), *Spirulina* (76%), and *Chlorella* (75%) [33]. Despite this high digestibility for SFA, the overall reduction in digestibility of protein, amino acids, lipid, fatty acids and energy in the *N*. *oculata* co-product, versus whole cells, may explain the lower weight gain at higher co-product inclusion levels.

Results of the 84-day growth experiment using *N*. *oculata* co-product showed that replacing 33% of fishmeal with *N*. *oculata* co-product achieved final weight, weight gain, percent weight gain, specific growth rate (SGR), and protein efficiency ratio (PER) that are comparable to the reference diet (Nanno0) but that higher inclusion levels lowered these fish performance measures. Higher inclusion levels of *N*. *oculata* co-product, however, did not depress feed conversion (FCR), feed intake (FI), and fish survival, given that we observed no significant differences among the diets. Also, tilapia showed high palatability to *N*. *oculata* co-product diets, feeding equally well on these diets as on the reference diet (Nann0, 7% fishmeal by weight).

These growth experiment results provide the first report on effects of inclusion levels of a microalgal co-product in tilapia feed. A few studies have examined microalgae co-products in feeds of other aquaculture species. Sørensen et al. (2017) [52] reported that the co-product of *Nannochloropsis oceania* can be used at modest inclusion levels, around 10%, without negative effects on the performance of Atlantic salmon. Kiron et al. (2012) [53] reported no reduction in weight gain of Atlantic salmon (*Salmo salar*) fed diets in which whole and lipid-extracted algal meals replaced 5 or 10% of dietary protein from fishmeal. The same authors reported that 25–40% protein replacement with two algal co-products (*Nanofrustulum* and *Tetraselmis*) was possible in common carp and *Litopenaeus vannamei*. Patterson and Gatlin (2013) [29] reported that co-product from *Nannochloropsis salina* could replace up to 10% of crude protein from fishmeal and soy protein concentrate without causing significant reductions in juvenile red drum performance. Our results and these prior studies suggest that lower trophic level aquaculture species (tilapia, carp, shrimp) perform better on higher dietary inclusion rates of micro-algal co-product than do higher trophic species (salmon, red drum).

Our findings highlight the need to better understand how anti-nutrients in microalgal co-product limit inclusion rates in aquafeeds and to identify practical steps to improve nutrient digestibility to achieve higher replacement levels. The observed reduction in nutrient digestibility could be due to the high content of complex indigestible cell wall non-starch polysaccharides (NSPs) in the microalga [54]. *Nannochloropsis* sp. cell walls are known to include complex polysaccharides [55]. Complex non-starch polysaccharides, such as cellulose, gums, pectins and hemicelluloses, are undesirable in aquafeed because they depress nutrient absorption [56,57,58]. Lipid extraction from *N*. *oculata* whole cells seems to elevate anti-nutrient levels including NSP in *N*. *oculata* co-product biomass, which might explain our results on *N*. *oculata* co-product digestibility and growth effects. Specifically, anti-nutrient analysis (Table 4) showed that *N*. *oculata* co-product contains higher levels of crude fiber (3.7%) than whole cells (0.8%), higher hemicellulose level (43.3 ugmg^−1^) than whole cells (28.8 ugmg^−1^), higher level of trypsin inhibitor (2145.0 TIU/g) than whole cells (1000TIU/g), and higher level of lectin (240.0 HU/mg) than whole cells (19.0 HU/mg). Finally, composition analysis indicated that neither *N*. *oculata* whole cells nor co-product contain pectin, which provides an advantage to formulate aquaculture feed with co-product. Pectin is a polysaccharide characterized by relatively high extractability using acid or chelators and a high content of D-galacturonic acid (GalUA) units as the backbone, connected in chains through a glycosidic linkage [59]. These uronic acids have carboxyl groups which, in the presence of divalent cations (usually calcium), have considerable effects on viscosity, solubility, and gelation formation [60].

Our results fit with several prior studies that revealed that NSPs have a negative influence on digestion and absorption by modulating digesta viscosity and rate of passage, and by altering fish gut physiology and morphology [36,61]. The presence of digestive enzymes that can hydrolyze the β-glycosidic bonds of NSP seems to be very low or nonexistent in monogastric animals including tilapia [48]. Thus, highly concentrated amounts of complex NSPs, and trypsin inhibitor content in *N*. *oculata* co-product could have inhibited nutrient digestibility in tilapia in our experiment. In our previous study, we also found depressed digestibility of fatty acids and amino acids in *Chlorella sp*. whole cells, likely due to their complex cell wall structure [33]. Similarly, Skrede et al. (2011) [62] revealed that the complex cell wall structure of algae may affect digestibility of inherent algae lipids and other lipids in the diets. As the digestive tracts of tilapia lack any appreciable NSP enzyme activity [48], adding NSP enzymes to feed could enhance digestibility and utilization of nutrients supplied by *N*. *oculata* co-product [63,64]. Also, extrusion processing of feeds containing *N*. *oculata* co-product would break down trypsin inhibitors and thus could improve the digestibility and retention of protein and lipids, and feed conversion in fish. Similarly, previous studies on plant protein have revealed that extrusion could enhance the utilization of protein, starch and NSPs [65,66,67]. We did not extrude the feeds used in our experiments and recommend doing so in future work with *N*. *oculata* co-product.

Consistent with many aquaculture studies [34], we found that dietary fatty acid composition was mirrored in the fatty acid profile and composition of edible fillet tissue of Nile tilapia. Similar levels of fish oil, total n6 PUFA and total n3PUFA in all formulated diets (Tables 6 and 7) resulted in similar levels of major n3 and n6 PUFA in fillets of fish across all the diets. This lack of difference in fillet composition suggests that tilapia utilized nutrients in the *N*. *oculata* co-product as efficiently as they used nutrients in fishmeal. Also, tilapia fed the Nanno100 diet for 84 days yielded 2.2 mg total EPA+DHA per g of raw fillet (Table 14), suggesting that *N*. *oculata* co-product would be a good candidate for EPA supplementation in tilapia diet formulation.

Our results also have bearing on the n3:n6 fatty acid ratio in a person’s total diet, which should be 1:1 or higher for optimum health. All diets yielded a n3:n6 ratio close to 1 in tilapia fillets. Thus, even full replacement of fishmeal with *N*. *oculata* co-product (Nanno100 diet) can still maintain a favorable ratio for the human consumer [34,68].

The limited published data on elemental composition of microalgae whole cells and co-product indicate that essential minerals (e.g., Ca, Mg, P, and K), and certain trace elements (e.g., copper and manganese) are generally found at higher levels in microalgae than terrestrial feed ingredients [69]. The levels we detected in *N*. *oculata* whole cells and co-product (Table 5) fit in the previously reported range for microalgae. We found similar levels for most macro minerals in the *N*. *oculata* co-product and whole cells; and lower levels of trace elements in the lipid-extracted *N*. *oculata* co-product than in whole cells (Table 5). Also, depositions of macro minerals and several trace elements in tilapia fillet were not significantly different among all dietary treatments (Table 12).

Trace element concentrations in fillets were lower than in the diets (Table 8), except for no change in selenium, suggesting these trace elements may be excreted by Nile tilapia. Aluminum (Al) and iron (Fe), for example, have been examined in a few dietary toxicity studies in tilapia [70,71]. We found that fillet concentrations, compared to diet concentrations, declined to below detection for Al and declined by an order of magnitude for Fe. Prior investigators reported that the Al and Fe taken up by tilapia were not accumulated in the body and subsequently excreted in the feces [70]. Feeding *Spirulina* containing high dietary level of Al (>2816 mg kg^-1^ diet) and Fe (>7573 mg kg^-1^ diet) as a sole protein source compromised the growth of juvenile tilapia [71]; but these dietary Al and Fe levels were an order of magnitude higher than in all our diets (Al <94.9 mg kg^-1^ diet, iron <133.6 mg kg^-1^ diet). Also, a clear advantage of using the *N*. *oculata* co-product over whole cells in aquafeeds is that Al and Fe levels were an order of magnitude less in co-product than in whole cells (Table 5). In another study, Hussein et al. (2014) [72] found no negative effect of high concentration of dietary Al (1792 mg kg^-1^ diet) and iron (3059 mg kg^-1^ diet) on growth and body composition of Nile tilapia in diet containing 38% algae; again, these levels were higher than in all our diets. Studies in other fish species also found no toxic effects of dietary Al concentrations of 100 to 2000 mg kg-1 [73,74]. We found low or non-detectable levels of other trace elements such as lead, mercury, and arsenic in the co-product, feeds, and fillet. We detected a low concentration of arsenic levels in the *N*. *oculata* co-product (0.2 mg kg^-1^), lower than in whole cells (5.9 mg kg^-1^), and arsenic levels were non-detectable in the experimental diets and fillets. We also detected a low concentration of lead (0.1 mg kg^-1^) in the *N*. *oculata* co-product and all diets containing the co-product. Lead is a highly toxic metal that causes health problems in many parts of the world; and the US Center for Disease Control considers lead poisoning to be the leading environmental health threat to children at 10μg/dL concentration in blood [75]. We found non-detectable levels of lead concentrations in fish fillets across all diets; thus, fish fed the fishmeal free diet, with 8% *N*. *oculata* co-product inclusion by weight, yielded a fillet which is safe eating for human health.

## Conclusion

Microalgae based diets are currently in the preliminary development stage for aquafeeds. Achieving wide use of such a nutrient dense *N*. *oculata* co-product in fish feeds requires researchers to find ways to enhance nutrient digestibility. Developing highly digestible algal ingredients will both improve feed conversion ratios and reduce nutrient loads in fish culture effluents, while also helping drive algae-based aquafeeds towards cost-competitiveness with conventional feed. Towards this goal, we are now focusing on whether the inclusion of one or more non-starch polysaccharide and protease enzymes in *N*. *oculata* co-product diet enhances nutrient digestibility and retention and growth and reduces effluent nutrient loading in Nile tilapia.

The aquafeeds industry will use microalgal co-product as a nutrient source only if it is a highly digestible ingredient that improves feed conversion ratios and reduce nutrient loads in fish culture effluents [11,33,34,35,76,77]. As we mentioned earlier, microalgal co-product also needs to become cost-competitive with conventional protein sources for aquaculture feeds. Fortunately, innovative business models by leading microalgae companies are emerging to meet this challenge [78]. These include multi-product “biorefinery” approaches, such as Cellana LLC’s current business model, which is anchored by high-value omega-3 supplements and high-value inks, allowing for flexible pricing of the microalgal co-products for aquafeeds and other commodity feed, food, and fuel applications (Martin Sabarsky, personal communication).
